# Draft Genome Sequences of *Streptococcus agalactiae* Serotype Ia and III Isolates from Tilapia Farms in Thailand

**DOI:** 10.1128/genomeA.00122-16

**Published:** 2016-03-24

**Authors:** Nontawith Areechon, Korntip Kannika, Ikuo Hirono, Hidehiro Kondo, Sasimanas Unajak

**Affiliations:** a Department of Aquaculture, Faculty of Fisheries, Kasetsart University, Chatuchak, Bangkok, Thailand; b Graduate School of Marine Science and Technology, Tokyo University of Marine Science & Technology, Minato-Ku, Tokyo, Japan; c Department of Biochemistry, Faculty of Science, Kasetsart University, Chatuchak, Bangkok, Thailand

## Abstract

*Streptococcus agalactiae* serotypes Ia and III were isolated from infected tilapia in cage and pond culture farms in Thailand during 2012 to 2014, in which pathogenicity analysis demonstrated that serotype III showed higher virulence than serotype Ia. Here, we report the draft genome sequencing of piscine *S. agalactiae* serotypes Ia and III.

## GENOME ANNOUNCEMENT

*Streptococcus agalactiae*, or group B streptococcus (GBS), is the Gram-positive pathogenic bacterium that affects a number of animal species, including fish. This bacterium is an important pathogen due to its frequent association with septicemia and meningoencephalitis (1, 2), and it has caused morbidity and mortality in fish aquaculture worldwide (3–5). To date, some genome sequences of *S. agalactiae* from fish isolates have been reported (2, 6–12). Most of the deposit sequences are recorded as serotype Ia, which is associated with beta-hemolytic and nonhemolytic strain serotype Ib (6, 12). From our study, *S. agalactiae* serotypes Ia and III, determined by *cps* cluster, were isolated from infected tilapia cultured in cages and ponds in the central, northern, northeastern, and southern part of Thailand during 2012 to 2014, and both serotypes exhibited beta-hemolytic activity. The pathogenicity of the two serotypes were compared by intraperitoneal injection in tilapia, which demonstrated that serotype III showed much higher virulence than serotype Ia. In this study, the first draft genome report of *S. agalactiae* serotypes Ia and III from tilapia are recorded.

The genomic DNAs from *S. agalactiae* serotypes Ia and III, strains JP9 and JP17, respectively, were isolated using the standard phenol-chloroform extraction method and applied for the library preparation using the Illumina Nextera XT DNA sample preparation kit (Illumina, USA). The samples were accessed for the genome sequence by the Illumina MiSeq platform with the MiSeq reagent kits, version 2 (300 cycles) (Illumina). Assembly of the raw reads was performed with CLC Genomics Workbench version 6.5.1, and then the scaffolds were analyzed on the RAST server (13). Multilocus sequence typing of the strains was performed using the *S. agalactiae* MLST database (http://pubmlst.org/sagalactiae/) (14).

By sequencing of the genomes, 3,591,791 and 2,549,795 reads from JP9 and JP17, respectively, were obtained. They are assembled into 58 and 34 contigs, comprising 2,105,692 and 2,041,480 bp, respectively. Using the RAST server, 2,094 and 2,076 coding regions were predicted for JP9 and JP17, respectively. By multilocus sequence typing, JP9 was determined to be a multilocus sequence type 7 strain (ST7), whereas JP17 was ST283, which is in accordance with a previous report (8).

### Nucleotide sequence accession numbers. 

The next-generation sequencing (NGS) reads were registered under the accession no. DRA004157, and the partial genome sequences of JP9 and JP17 have been deposited in DDBJ/EMBL/GenBank under the accession numbers BCNI01000001 to BCNI01000058 and BCNJ01000001 to BCNJ01000034, respectively.
